# Pancreatic β-Cell Identity Change through the Lens of Single-Cell Omics Research

**DOI:** 10.3390/ijms25094720

**Published:** 2024-04-26

**Authors:** Floris Leenders, Eelco J. P. de Koning, Françoise Carlotti

**Affiliations:** Department of Internal Medicine, Leiden University Medical Center, 2333 ZA Leiden, The Netherlands; f.leenders@hubrecht.eu (F.L.); e.dekoning@lumc.nl (E.J.P.d.K.)

**Keywords:** diabetes mellitus, β-cell identity change, β-cell dedifferentiation, β-cell heterogeneity, β-cell subpopulations, single-cell omics research

## Abstract

The main hallmark in the development of both type 1 and type 2 diabetes is a decline in functional β-cell mass. This decline is predominantly attributed to β-cell death, although recent findings suggest that the loss of β-cell identity may also contribute to β-cell dysfunction. This phenomenon is characterized by a reduced expression of key markers associated with β-cell identity. This review delves into the insights gained from single-cell omics research specifically focused on β-cell identity. It highlights how single-cell omics based studies have uncovered an unexpected level of heterogeneity among β-cells and have facilitated the identification of distinct β-cell subpopulations through the discovery of cell surface markers, transcriptional regulators, the upregulation of stress-related genes, and alterations in chromatin activity. Furthermore, specific subsets of β-cells have been identified in diabetes, such as displaying an immature, dedifferentiated gene signature, expressing significantly lower insulin mRNA levels, and expressing increased β-cell precursor markers. Additionally, single-cell omics has increased insight into the detrimental effects of diabetes-associated conditions, including endoplasmic reticulum stress, oxidative stress, and inflammation, on β-cell identity. Lastly, this review outlines the factors that may influence the identification of β-cell subpopulations when designing and performing a single-cell omics experiment.

## 1. Introduction

The main hallmark in the development of both type 1 diabetes (T1D) and type 2 diabetes (T2D) is a decline in functional β-cell mass [1,2]. Reduced β-cell mass is primarily caused by β-cell death, but growing evidence indicates that the loss of β-cell identity contributes to β-cell failure. During this process, β-cells lose both their phenotype and function, as indicated by a decreased expression of key β-cell identity markers and altered expression of genes involved in glucose-stimulated insulin secretion [3,4,5]. Dedifferentiation typically involves two features: (i) the downregulation of β-cell signature genes, including key transcription factors, insulin, and glucose metabolism-related genes; and (ii) the concomitant upregulation of genes that are otherwise suppressed or expressed at very low levels in mature β-cells, such as endocrine progenitor cell genes. Another form of identity change is transdifferentiation, in which a differentiated cell type is converted into another cell type upon the induction of a new set of signature genes.

In recent years, the field of single-cell omics research has expanded rapidly. The development of multiplexed assays has provided researchers with the tools to accurately and comprehensively describe cellular and molecular states in health and disease down to the single-cell level [6]. Especially in the endocrine pancreas, with its variety of cell types with different features, individual cellular changes are often overlooked or diluted when analyzed at the level of the whole islet or when examining cell populations in bulk. Single-cell omics techniques have great potential to overcome this issue.

**Figure 1:**
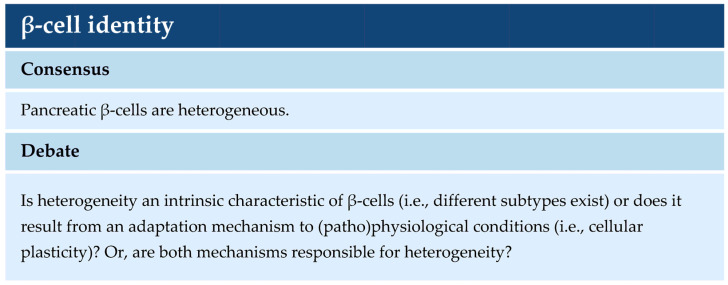
Current debate on β-cell heterogeneity.

In this review, we will first discuss recent single-cell omics research focused on β-cell identity in health, highlighting the observation of β-cell heterogeneity and the characterization of β-cell subpopulations in individuals without diabetes mellitus. The current consensus and the most prominent questions are presented in Figure 1. Then, we will compare these data to observations in patients with diabetes mellitus. Finally, we will shed light on the molecular mechanisms leading to β-cell identity change, emphasizing endoplasmic reticulum stress, oxidative stress, and inflammation.

## 2. β-Cell Identity

Pancreatic β-cells are the endocrine cells that synthesize, store, and release insulin. Together with glucagon, insulin is a key hormone to maintaining circulating glucose concentrations within a narrow physiological range. Although the cellular composition of the islets of Langerhans is heterogeneous, including α, β, δ, γ/PP, and ε endocrine cells, β-cells have long been thought to be a homogeneous cell type. However, in the past, there have been hints of functional heterogeneity within β-cells [7]. We also found that there are regional functional differences in β-cell adaptation, in which mouse islets from the splenic region of the pancreas and the tail are more responsive to high-fat diet-induced insulin resistance than islets in the duodenal and gastric regions [8]. Likewise, individual rat β-cells react differently in terms of insulin secretion in response to the same glucose stimulation in vitro, ranging from a secreting to a non-secreting state and with varying states in between [9]. Similar to mouse and rat β-cells, it is suggested that human β-cells are highly heterogeneous in terms of insulin secretion in a way that a small fraction of β-cells contributes to the majority of insulin secreted [10]. By combining large-scale functional cell mapping with optogenetics and photopharmacology, Johnston et al. proposed a revised blueprint for islet function whereby a few pioneering β-cell hubs dictate emergent population behavior in response to glucose in isolated mouse islets [11]. The ‘hub cells’ display a lower expression of nk6 homeobox 1 (Nkx6.1), pancreatic and duodenal homeobox 1 (Pdx1), and insulin (Ins) compared to the rest of the β-cell population. The possibility that β-cell hubs represented a multihormonal or progenitor population was ruled out by the fact that there was no co-localization with glucagon and neurogenin 3, respectively. According to the researchers, hub cells constitute a metabolically adapted, repurposed subpopulation of β-cells that display features of immature cells. Along the same line, Salem et al. described both in zebrafish and in mice a population of ‘regulatory β-cells’ within coordinated islet networks in vivo, driving Ca^2+^ dynamics and insulin secretion [12]. A possible reason for the existence of β-cell subpopulations in terms of functionality could be that the dissimilarities between β-cell subsets may serve to create smooth rather than sharp changes in insulin secretion in response to various stimuli and to ensure that rapid shifts in blood glucose levels do not occur.

In line with functional heterogeneity, there is also evidence of β-cell heterogeneity regarding the expression of key genes. In islet cells isolated from rats, β-cell subpopulations with high glucose responsiveness express an abundance of a polysialylated form of neural cell adhesion molecule, or PSA-NCAM [13], as well as E-cadherin [14]. In mouse islets, Bader et al. reported that flattop (*Fltp*), a wnt/planar cell polarity effector and reporter gene, acts as a marker gene that subdivides mouse endocrine cells into two subpopulations and distinguishes proliferative from mature β-cells with distinct molecular, physiological, and ultrastructural features [15]. Additionally, *Fltp* knockout mice showed small but significantly reduced basal glucose and insulin levels, indicating that this marker gene is also functionally involved in the insulin secretory machinery. In isolated human islets, subsets of β-cells are found with high expression levels of dickkopf wnt signaling pathway inhibitor 3 (DKK3), a secretory modulator of canonical wnt/beta catenin signals, which is involved in the control of stem cell proliferation, homeostasis, and differentiation [16]. Also, vesicular monoamine transporter 2 (VMAT2), which plays an important role in the regulatory mechanism of insulin release, is expressed in a particular subset of human β-cells [17]. In a single-cell mass cytometry analysis, Wang et al. found three major clusters of β-cells in human islets [18]. Proliferating β-cells occupy two of these three clusters and are inversely correlated with age, leading the researchers to propose that with age, some of the β-cells switch from a proliferative state to a more quiescent state.

The emergence of single-cell omics techniques has greatly expanded our knowledge about islet cell subpopulations and their molecular, genetic, and functional features. By applying genomics, transcriptomics, and proteomics, traditional techniques can be complemented, and different subsets of β-cells and their identities can be described in more detail. Using a single-nucleus assay for transposase-accessible chromatin sequencing, or snATAC-seq, it has been shown in murine pancreatic islets that both α- and δ-cells appear poised, but repressed, from becoming β-cells, supporting earlier findings on the plasticity in identity of non-β-cells under certain circumstances [19]. Zeng et al. identified two subpopulations of β-cells in mouse islets: one with high mitochondrial membrane potential, representing a rare immature population with high proliferative capacity; and one with upregulated oxidative phosphorylation genes, postnatally representing cells with a mature insulin secretory response [20]. Focusing on maturity-specific features in mouse islet cells, van der Meulen et al. described a population of immature β-cells that is present throughout life and forms from non-β-cell precursors at a specialized micro-environment or ‘neogenic niche’ at the islet periphery [21]. These β-cells lack the expression of the late maturation marker urocortin 3 (UCN3) and are transcriptionally immature, lack cell-surface glucose transporter 2 (GLUT2), cannot sense glucose, and do not support calcium influx in response to depolarization. According to the researchers, however, the existence of this particular β-cell subset does not correspond with subpopulations previously described that are based on hub features [11] or the expression of *Fltp* [15] because the β-cell volume, location within the islet, and expression of UCN3 do not match these studies. Focusing on the epigenome, two major subtypes β-cells were described based on histone mark heterogeneity, termed β_HI_ and β_LO_, by Dror et al. [22]. Both subsets differ in size, morphology, cytosolic and nuclear structure, epigenomes, and cell surface marker expression. Functionally, β_HI_ cells have increased mitochondrial mass, activity, and insulin secretion in vivo and ex vivo. Interestingly, both subtypes were conserved in humans, with β_HI_ cells enriched in individuals with T2D.

In addition to their occurrence in mouse islets, β-cell subpopulations are also described through single-cell omics research in human islets. Dorrell et al. reported the identification of four distinct human β-cell subpopulations based on the presence of cell surface markers ST8S1A1 and CD9 [23]. Transcriptome analysis by RNA sequencing indicated that all four populations clearly displayed the gene expression profile of classical β-cells with very high levels of insulin mRNA and other typical marker genes, such as *PDX1*, *NKX6.1*, and MAF BZIP transcription factor A (*MAFA*). However, a subset of genes was consistently expressed at different levels in the β-cell subtypes. Some of these differentially expressed genes have been associated with insulin secretion, such as *GLUT2* [24]. Indeed, glucose-stimulated insulin secretion differed between the β-cell subpopulations. However, the genes for the aforementioned human heterogeneity markers DKK3 and VMAT2 were not significantly differentially expressed in these β-cell subsets. Using single-cell transcriptomic data, Segerstolpe et al. described five subclusters of β-cells with the combinatorial expression of the transcriptional regulators retinol-binding protein 4 (*RBP4*), free fatty acid receptor 4 (*FFAR4*), and inhibitor of DNA binding 1 (*ID1*), 2 (*ID2*), and 3 (*ID3*) [25]. *RBP4*, an adipokine primarily expressed in the liver and adipocytes [26], was found to be expressed in two of these five clusters, as well as in δ-cells. In obese and T2D individuals, increased circulating levels of RBP4 are elevated [27]. Additionally, agonists of *FFAR4*, which were expressed in the same clusters of *RBP4*-expressing β-cells, have been shown to induce insulin release in mouse pancreatic islets [28]. On the other hand, we identified subpopulations of human β-cells expressing higher levels of the gene ferritin heavy chain 1 (*FTH1*) [12], which encodes the heavy subunit of ferritin, the major intracellular iron storage protein, which is implicated in response to oxidative stress [29]. Similarly, Baron et al. found a cluster of human β-cells with differentially expressed endoplasmic reticulum stress-inducible genes homocysteine-inducible ER protein with ubiquitin like domain 1 (*HERPUD1*), heat shock protein family A member 5 (*HSPA5*), and DNA damage-inducible transcript 3 (*DDIT3*). However, it cannot be ruled out that this heterogeneous stress signature in β-cells reflects a variable response to pancreatic isolation, transport, and/or islet cell dissociation [30]. β-cell subtypes can also be distinguished by chromatin activity, as Chiou et al. showed using snATAC-seq [31]. The researchers found that heterogeneity in the β-cell epigenome mapped to cellular states related to insulin production and to a stress-related signaling response. More specifically, β-cells segregated into an *INS*-high and an *INS*-low population. Genes with increased promotor accessibility in hormone-high states were enriched for hormone secretion and glucose response, while genes with increased promotor accessibility in hormone-low states were enriched for a stress-induced signaling response. In a follow-up study from the same group, the presence of the two distinct β-cell subtypes was confirmed, with the majority subtype being maintained by core transcription factors hepatocyte nuclear factors 1 alpha (*HNF1A*), 4 alpha (*HNF4A*), and 4 gamma (*HNF4G*), while the minority subtype was maintained by transcription factor 4 (*TCF4*), neuronal differentiation 1 (*NEUROD1*), and nuclear factor I A (*NFIA*), all of which are associated with insulin secretion [32]. Using scRNA-seq, Tritschler et al. identified six β-cell clusters, which did not form separate clusters but rather connected states in the continuous β-cell manifold [33]. The largest cluster was annotated as mature β-cells, highly expressing canonical β-cell identity and maturity genes, while two smaller clusters were associated with genes involved in stress- and apoptosis-related pathways. The state in between the mature and stress-related clusters mostly resembled immature β-cells. In a single-nucleus RNA-seq setup, three subclusters of β-cells were distinguished: an *INS* pre-mRNA cluster, an intermediate *INS* mRNA cluster, and an *INS* mRNA-rich cluster, displaying distinct gene expression patterns representing different biological states [34]. Finally, a subset of β-cells was described by Rubio-Navarro et al. that was marked by high CD63 expression [35]. This β-cell subset is enriched for the expression of mitochondrial metabolism genes and exhibits higher mitochondrial respiration compared with CD63-low β-cells.

In some cases, the distinction between β-cell subpopulations is less pronounced, or to a higher degree dependent on maturation- or location-related features. In mouse islets, it was shown that there is some degree of β-cell heterogeneity at the early stage of islet development in terms of different maturation states [36]. However, during the process of development, the maturation states of these β-cells became synchronized and β-cell heterogeneity was not identified anymore in adult mouse islets. A recent paper showed two β-cell populations in mouse islets that appear to respond differently to a proinflammatory cytokine treatment. However, the authors showed that this differential sensitivity relied solely on the location of the cells within the islet rather than on the existence of two separate β-cell subsets [37]. Furthermore, in human islets, there have been multiple transcriptomic studies where no β-cell heterogeneity or existence of β-cell subpopulations was reported [38,39,40]. 

In conclusion, there are multiple reports on the existence of β-cell subpopulations in both human and mouse pancreatic islets, as summarized in Table 1. However, these studies often use different criteria to cluster β-cells into particular subsets, with some papers focusing on functional capacity while others highlight transcriptional or genomic features. But even between studies with a seemingly similar outset, there appears to be some discrepancy in the features of β-cell subsets identified, while other studies do not report any significant differences between β-cells. There could be several reasons for these contrasting reports on the identification of β-cell subpopulations. First of all, single-cell transcriptomics has limited sensitivity as opposed to the robust, but more crude, bulk methods. Technical artifacts or techniques with limited sensitivity may compromise the exploration of distinct differential expressions of gene or protein markers. Regarding scRNA-seq studies, for example, differential expression analysis has been proven to be a somewhat restricted tool for identifying alterations in biological processes between two experimental groups in small-scale studies with heterogeneous starting material [41]. Another crucial aspect is the number of cells analyzed. Some early studies may have processed too few cells to be able to pick up subpopulations of islet cells, especially if those cells are identified by lowly expressed genes. The molecular profiling of different clusters may also be influenced by donor variability, the effects of islet isolation procedures, and the pancreatic region. We will return to this discussion point at the end of the review. 

Overall, although single-cell omics methods still have to be further optimized to cope with the caveats mentioned above, they do have the potential to unravel β-cell subtype-specific transcriptomic, epigenomic, and functional features. Next, we will explore how changes identified in the gene regulatory programs of β-cell subpopulations might drive the pathogenesis of diabetes.

## 3. β-Cell Identity in Diabetes

Diabetes is caused by the absolute or relative insufficient production of insulin. Until recently, a reduced β-cell mass had been ascribed primarily to β-cell death. However, an increasing amount of evidence indicates that alterations in β-cell identity could contribute to the decreased function of the remaining β-cells. For the past decade, however, β-cell identity change has been proposed as a process contributing to the declined functional β-cell mass that is characteristic of diabetes [3,4,5]. First proposed as a mechanism underlying β-cell failure in mice by Talchai et al. [3] and as reviewed by Bensellam et al. [47], β-cell dedifferentiation entails the process of β-cells reverting to a dedifferentiated status. In humans, descriptive evidence of alterations in β-cell identity that are found in pancreatic islets from individuals diagnosed with T2D is limited, including an increased frequency of polyhormonal cells, a reduced expression of key β-cell transcription factors such as MAFA and PDX1, and an increased proportion of degranulated islet cells [48,49,50]. In isolated human islets, we reported the downregulation of β-cell maturity markers, particularly MAFA, in a model of drug-induced diabetes [51]. Additionally, the disruption of primary human islet integrity triggers severe phenotypic alterations in β-cells including the conversion of part of the insulin-producing β-cell population into glucagon-positive α-cells, an example of the aforementioned transdifferentiation [52]. Collectively, these findings point toward a link between the loss of β-cell identity change and reduced functional β-cell mass. Single-cell omics methods can be used to further investigate this link and to characterize the molecular mechanisms that might be at the basis of β-cell failure in diabetes.

In one of the first single-cell transcriptomics studies performed in human islets derived from T2D individuals, Wang et al. found that β-cells from T2D donors display a more immature gene signature with features seen in children, indicating at least a partial dedifferentiation process [38]. Upregulated genes in T2D β-cells were involved in cell cycle regulation programs and insulin secretion, suggesting that endocrine cells in T2D individuals are not able to maintain a fully differentiated gene expression profile. However, a high degree of gene expression variability within a given endocrine cell type was found and the number of total cells was too low to control for inter-individual differences and to profile rare, mixed-signature cells. In a follow-up study, the total number of processed cells was significantly increased, and the researchers confirmed that a large fraction of T2D β-cells acquire an immature gene expression profile [42]. This profile was characterized by the de-repression of juvenile gene sets and the activation of genes that are typically expressed in the exocrine compartment. Importantly, no upregulation of progenitor markers, such as neurogenin 3 (*NEUROG3*), POU class 5 homeobox 1 (*POU5F1*), and nanog homeobox (*NANOG*) was found in T2D β-cells, in contrast to what was previously reported in diabetes mouse models [3]. Xin et al. showed that 48 transcripts were differentially expressed between β-cells from islets isolated from T2D donors and donors without diabetes [39]. Interestingly, only a small fraction of these genes has been associated with T2D or has been reported to modulate islet cell function or growth. Segerstolpe et al. found that there were significantly lower *INS* mRNA levels in T2D β-cells compared to those in healthy individuals [25]. Gene set enrichment analysis showed that most cell types in T2D individuals displayed significantly downregulated genes responsible for energy metabolism in mitochondria and protein synthesis, while genes responsible for apoptosis, diabetic nephropathy, and cytokine signaling were upregulated. In a multiomics study conducted by Wigger et al., pancreatic tissue samples were collected from metabolically phenotyped pancreatectomized individuals, including donors without diabetes, donors with impaired glucose tolerance (pre-diabetes), and T2D donors [43]. The authors reported that islets from pre-diabetes and T2D individuals showed an upregulation of islet genes that were functionally related to cell–extracellular matrix interactions, immune responses, and signaling pathways. In contrast, gene expression related to RNA processing, protein translation, and mitochondrial oxidative phosphorylation was downregulated compared to individuals without diabetes, similarly to the Segerstolpe study [25]. The strength of the enrichment increased with the progression of the disease, suggesting that early dysregulation of gene pathways worsens with the decline in β-cell function. Looking at the proteomic profiles, the researchers showed that data points from donors without diabetes clustered closely, indicating a very similar proteome signature, while those of T2D donors revealed substantial proteome heterogeneity among each other. Aldolase fructose-biphosphate B (ALDOB), a marker of β-cell precursors, was upregulated in pre-diabetes and T2D donors as compared to donors without diabetes, indicating that mature β-cells may have reverted to a more immature stage of differentiation. In contrast, there was no upregulation of markers of immature β-cells, β-cell precursors, or other islet cell types, while key determinants of mature β-cells, such as *PDX1*, *MAFA*, *NKX6.1,* or *UCN3* were unchanged at the transcriptomic level. In addition to the Segerstolpe and Wigger studies mentioned above [25,43], in a similar analysis but on a different set of donors, Lawlor et al. found similar significantly induced or repressed genes in pancreatic islets from both donors without diabetes and T2D donors when comparing single-cell RNA-seq data [40]. However, the researchers did not identify significant shifts in islet cell populations, increases in the number of hormone-negative cells, or other evidence of β-cell dedifferentiation or transdifferentiation in T2D islets. Using integrative transcriptomic and epigenomic analysis, Weng et al. showed that *HNF1A* expression was reduced in β-cells from T2D donors [44]. Heterozygous mutations in *HNF1A* are sufficient to cause the most frequent form of the maturity onset diabetes of the young (MODY) [53]. Because of these findings, along with reports stating that common variants at the *HNF1A* locus have been associated with T2D [54], the authors propose a causal role for *HNF1A* regarding β-cell heterogeneity in T2D pathogenesis. Focusing on T1D and making use of the Human Pancreatic Analysis Program (HPAP) [55,56,57], Fasolino et al. found that Aab+ donors (individuals with autoantibodies toward pancreatic islet proteins but no clinical diagnosis of T1D) exhibit similar transcriptional changes as T1D donors in various endocrine cells, despite the first group of donors retaining normoglycemia [45]. In contrast, Elgamal et al. did not observe significant transcriptional changes in Aab+ donors from the HPAP initiative, but there was a marked upregulation of processes related to MHC class 1 antigen presentation and cytokine signaling [46]. In data retrieved from T2D donors, the authors observed a downregulation of genes involved in mitochondrial function and other processes implicated in β-cell function.

In summary, a growing body of evidence indicates that the loss of β-cell identity occurs in diabetes, with some degree of dedifferentiation to a more immature state, as shown in Table 1. In addition, several studies reported that protein synthesis machinery, as well as pathways related to mitochondrial metabolism, are affected in islets from donors with diabetes. However, there are also some discrepancies. Just a few studies show an increase in progenitor markers and, thus, a potential return to a β-cell precursor state, for example. One of the possible confounding factors could be the difficulty in discerning actual biological, diabetes-related effects on β-cell identity from effects that occur due to differences in donor characteristics (genetic and environmental factors, including clinical situation before tissue procurement), procurement aspects such as warm and cold ischemia time, donation after cardiac or brain death in cases of deceased organ donors, and tissue preparation (islet isolation and islet dispersion with or without sorting in case of single-cell omics analysis on isolated islet cells). Regarding organ donors, it is known that islets experience stress through macrophage infiltration after brain death, including activated components of apoptotic and inflammatory pathways [58,59]. In addition, the enzymatic digestion-mediated isolation of pancreatic islets and ex vivo culture can further change their molecular profile. Negi et al. reported the downregulation of several pancreas-specific transcription factors in cultured islets, while pancreatic progenitor cell-specific transcription factors like SRY-box transcription factor 9 (*SOX9*) are upregulated, potentially giving the false impression of identity change as a result of diabetes-related metabolic stress [60]. Furthermore, single-cell omics data derived from human donor material has been largely obtained from pancreatic islets isolated from deceased organ donors. These subjects are often classified according to a binary division into individuals with or without diabetes, rather than on a continuous glycemic scale, from euglycemia to hyperglycemia. Furthermore, diabetes is a heterogeneous condition, not only between different types, e.g., T1D, T2D, MODY, but also within each disease. In T2D, different degrees of insulin resistance and β-cell dysfunction exist which may obfuscate subtle changes in the β-cell phenotype when analyzed together. An underlying issue is the fact that researchers in general do not have access to extensive clinical and laboratory information about organ donors [61]. A more standardized approach with better access to donor history, combined with the intricate layering of single-cell multi-omics techniques, would enable researchers to acquire a more accurate and biologically truthful cross-sectional overview of β-cell identity change under pathophysiological conditions.

## 4. Endoplasmic Reticulum Stress

**Figure 2:**
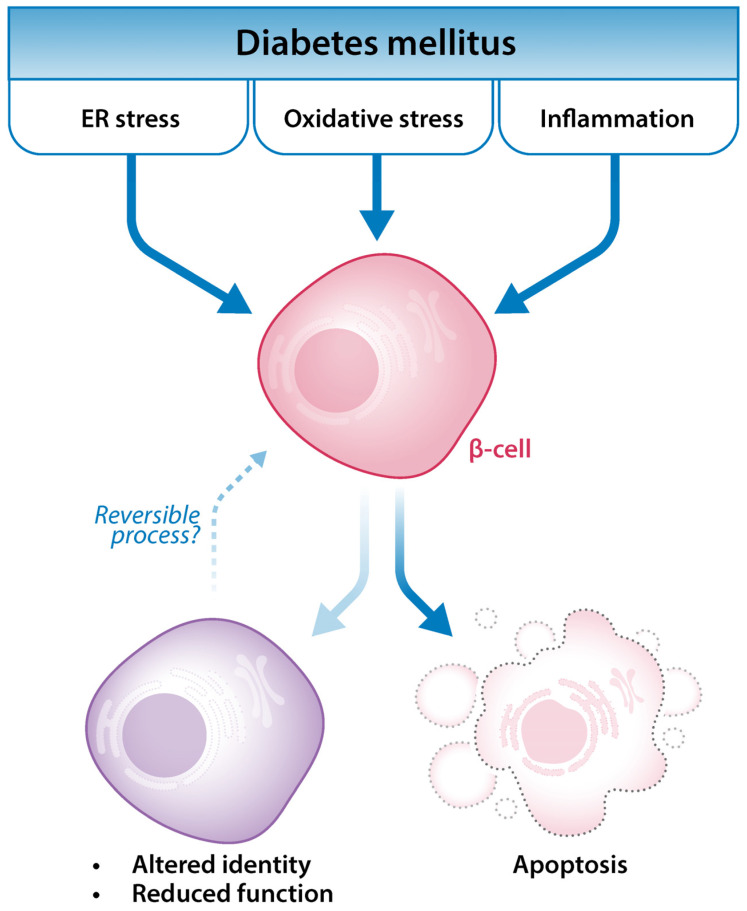
ER (endoplasmic reticulum) stress, oxidative stress, and inflammation are among the main biological processes underlying the development of diabetes mellitus. They may lead to β-cell death (apoptosis) but also to altered β-cell identity and function.

Among the molecular mechanisms underlying the complex etiology of the multifactorial disease that is diabetes mellitus, enhanced endoplasmic reticulum (ER) stress has been increasingly acknowledged (Figure 2). ER stress results from the accumulation of unfolded or misfolded proteins within the ER. It typically occurs when an increased demand for proteins overloads the ER’s folding mechanism, which causes unfolded proteins to build up in the lumen. In addition to protein overload, exposure to environmental toxins, viral infections, inflammatory cytokines, mutant proteins, and aging can also induce ER stress [62]. ER stress is part of a protective mechanism to preserve or restore β-cell health by re-establishing homeostasis and regaining normal ER function. However, under irresolvable ER stress, the unfolded protein response (UPR) switches from an adaptive to an apoptotic role, potentially leading to reduced β-cell mass in diabetes [63]. In addition, exacerbated ER stress and a prolonged UPR are implied in the decline of β-cell function. The overexpression of the active form of X-box binding protein 1 (XBP1), a downstream target of the UPR, impairs glucose-stimulated insulin secretion [64]. In addition, a proteomic study comparing the insulin secretion of glucose-responsive and non-responsive insulinoma cell lines identified lower levels of UPR-related chaperone proteins such as glucose-regulated protein 78kDa (GRP78), glucose-regulated protein 90kDa (GRP94), protein disulfide isomerase (PDI), and endoplasmic reticulum protein 29 (ERP29) in non-responsive cells, suggesting that the depletion of such proteins may affect β-cell function [65]. 

Furthermore, ER stress has been linked to β-cell identity loss. By comparing time-dependent gene expression changes in islets of diabetes-prone (db/db mice) and diabetes-resistant (ob/ob mice) mouse models of obesity, the failure of the adaptive UPR was seen in diabetic mice [66]. This process was associated with a downregulation of key β-cell transcription factors and other β-cell-enriched genes in diabetic db/db mice, which was partially restored upon treatment with the chemical chaperone 4-phenylbutyrate. The absence of the PERK-mediated phosphorylation of eukaryotic initiation factor 2 on Ser51 of the α subunit (eIF2α) leads to a decreased expression of the β-cell-specific transcription factors *Pdx1* and *Mafa* in murine β-cells [67]. In the human β-cell line EndoC-βH1, ER stress leads to disrupted expression of the key β-cell fate- and function-related genes forkhead box O1 (*FOXO1*) and nk2 homeobox 2 (*NKX2.2*) [68]. In pancreatic sections of donors with T1D, an increased frequency of chromogranin-positive, hormone-negative cells was reported [69]. In pancreatic sections of donors with T2D, ER stress-mediated β-cell apoptosis was associated with high expression rates of islet amyloid polypeptide [70], which we showed is associated with β-cell identity loss [49].

Focusing specifically on single-cell omics research, the link between ER stress and the loss of β-cell identity is further established. We have shown that the activation of ER stress is associated with the loss of key β-cell transcription factors [71]. MIN6 cells, exposed to reversible, chronic ER stress conditions, undergo transcriptional and translational reprogramming associated with the impaired expression of regulators of β-cell function and identity [72]. This reprogramming was reversed upon recovery from stress, indicating a high degree of adaptive plasticity, which is lost when stress episodes exceed a certain threshold. In mouse islets, the inactivation of TATA-box-binding protein-associated factor 4 (Taf4), a subunit of the general transcription factor II D (TFIID), was associated with an ER stress response and impacted the expression of critical genes involved in β-cell function leading to increased glycemia, lowered plasma insulin levels, and defective glucose-stimulated insulin secretion [73]. Moreover, one week after Taf4 loss, single-cell RNA-seq revealed cells with mixed β-, α-, and/or δ-cell identities as well as a β-cell population transdifferentiating into α-like cells. However, this proposed association between the occurrence of ER stress and alteration in β-cell identity is not always found. In a single-cell RNA-seq study conducted by Xin et al., the researchers found no evidence of dedifferentiation among human β-cells from different states with varying degrees of UPR activation [74]. Markers of β-cell dedifferentiation, including *NEUROG3*, *NANOG*, and *POU5F1*, were absent, and key β- and α-cell genes were unchanged.

Finally, dedifferentiation might be a way for β-cells to escape from death. In the context of T1D, modulating the ER stress response in β-cells of non-obese diabetic (NOD) mice by deleting the UPR sensor inositol-requiring protein 1 alpha (IRE1α) before insulitis induced the transient dedifferentiation of β-cells, resulting in substantially reduced islet immune cell infiltration and β-cell apoptosis [75]. Similarly, in the context of T2D, when comparing two obese mouse strains with different diabetes susceptibility given a diabetogenic diet, it was shown that the islets of the diabetes-resistant mouse strain developed into a ‘protective β-cell cluster’ with indications of reduced β-cell identity [76]. In contrast, β-cells of the diabetes-prone mouse strain responded with expression changes indicating metabolic pressure and ER stress, presumably leading to later β-cell loss.

In conclusion, the concept that enhanced ER stress plays a detrimental role in β-cell health during diabetes progression is well-established. Emerging evidence, coming from single-cell omics studies in particular, reveals that increased ER stress can also lead to alterations in β-cell identity. Nevertheless, some of these findings come from in vitro studies using pharmacological ER stressors; thus, further validation in vivo is needed. The question remains of what exactly is tipping the scale from ER stress being a protective mechanism to help β-cells cope with metabolic changes to ER stress having a detrimental effect on β-cell identity and contributing to β-cell failure in diabetes.

## 5. Oxidative Stress

Like ER stress, oxidative stress plays a pivotal role in the development of diabetes. Oxidative stress results from an imbalance between the production of reactive oxygen species (ROS) and the activity of the antioxidant defense system [77]. This imbalance causes disturbances in the normal redox state of cells, which in turn leads to toxic effects through the production of free radicals and peroxides that damage all components of the cell. The main source of ROS in the cell are the mitochondria, the powerhouse organelles that play a vital role in energy metabolism and the biosynthesis of amino acids, nucleic acid, and lipids [78]. Mitochondria are the only organelles in cells, besides the nucleus, that contain their own DNA. Mitochondrial DNA is thought to be particularly susceptible to ROS attack associated with oxidative stress, leading to mutations in the mitochondrial genome and further mitochondrial dysfunction [79]. Murine β-cells, but also human β-cells, are sensitive to ROS because they are low in antioxidant enzymes such as catalase, glutathione peroxidase, and superoxide dismutase [80,81,82,83]. It has long been proposed that oxidative stress plays a major role in the pathophysiology of both type 1 and type 2 diabetes [84,85,86,87]. In patients with the early onset of T1D, plasma levels of oxidative stress markers such as malondialdehyde and protein carbonyl groups are elevated and are even higher in early adulthood [88]. Biomarker levels in T2D patients show elevated antioxidant enzyme activity, lipid peroxidation, and nucleic acid oxidation compared to individuals without diabetes [89]. Similarly, T2D patients have higher total superoxide dismutase activity and an increased rate of lipid peroxidation compared to individuals without diabetes [90]. Additionally, the levels of nucleic acid oxidation markers 8-oxo-7,8-dihydroguanosine (8-oxoGuo) and 8-oxo-7,8-dihydro-2’-deoxyguanosine (8-oxodG) are increased in T2D patients compared to healthy individuals, while the level of 8-oxoGuo in urine independently predicts diabetes-related mortality, further establishing the link between the pathophysiology of diabetes and the occurrence of oxidative stress [91,92]. 

Oxidative stress has also been linked to β-cell identity loss. We showed that oxidative stress leads to the loss of β-cell function in human islets that is associated with an ER stress response and evidence of dedifferentiation, as indicated by the loss of β-cell maturity markers *MAFA* and *PDX1*, and the upregulation of endocrine progenitor markers *SOX9* and HES family BHLH transcription factor 1 (*HES1*) [93]. The decreased β-cell function upon the occurrence of oxidative stress was in line with studies performed in rodent β-cell lines, in which antioxidant treatment prevented the downregulated insulin gene expression that followed supraphysiological concentrations of glucose, suggesting the involvement of oxidative stress in β-cell failure [94]. The oxidative stress-induced loss of β-cell maturity genes has been documented in rodent β-cell lines and rat islets through the reduced expression of key β-cell transcription factors *Pdx1*, *Nkx6.1*, and *Mafa* [48,95,96,97]. Furthermore, reduced nuclear MAFA expression found in the islets of diabetic db/db mice is restored by the overexpression of the antioxidant enzyme endogenous glutathione peroxidase-1 (GPX1) [48,98]. Similarly, decreased nuclear PDX1 and MAFA expression in the islets of diabetic ZDF rats is prevented by treatment with the GPX mimetic ebselen [99]. Besides the downregulation of β-cell markers, an upregulation of progenitor markers could also be a sign of altered β-cell identity. In rat pancreatic islets treated with hydrogen peroxide, there was an increased expression of MYC proto-oncogene (C-MYC) [100], a transcription factor known to inhibit β-cell dedifferentiation [101,102]. In human islet cells cultured for 3 days, the upregulated expression of oxidative stress-related genes was associated with the increased expression of pancreatic progenitor cell-specific transcription factors such as *SOX9*, *SOX4*, and *ID2* [60].

Zooming in on single-cell omics, the link between oxidative stress and reduced β-cell function, mass, and/or identity is further established. In an integrated dataset of single-cell transcriptomics from three studies of β-cells derived from T2D individuals, the occurrence of oxidative stress was associated with decreased *INS* expression [103]. β-cells from T2D donors had normal *INS* expression levels under conditions of low oxidative stress, while β-cells displaying higher levels of oxidative stress markers showed decreased *INS* expression. In a similar approach, an integrated transcriptomic dataset comparing β-cells from T2D patients and individuals without diabetes revealed a set of oxidative stress-related genes that were differentially upregulated specifically in the T2D group [104]. To gain insight into the molecular events underlying β-cell adaptive flexibility in β-cells from db/db mice, a commonly used model of T2D, a combined proteomic and post-translational modification-specific proteomics (PTMomics) approach was applied [105]. By comparing the islets from db/db mice and wild-type controls, the researchers identified differential modifications of proteins involved in redox homeostasis and renin-angiotensin-aldosterone signaling (RAAS) associated with β-cell adaptive flexibility. RAAS is a biological process associated with the development of obesity-linked T2D, and its malfunctioning has been suggested to lead to insulin resistance by increasing cellular oxidative stress and impaired glucose metabolism in obesity and T2D [106,107,108]. In rats fed with a long-term high-fat, high-fructose (HFHF) diet to mimic hyperglycemia, plasmatic oxidative markers and nucleic acid oxidation markers were significantly increased, while single-cell RNA-seq indicated that islets of hyperglycemic rats had a downregulated expression of genes related to glucose-regulating hormone synthesis [109]. Moreover, oxidoreductase gene expression was upregulated in α-, β- and PP-cells from rats with the long-term HFHF diet, indicating that hyperglycemia accelerated oxidative stress in these islet subpopulations. More recently, RNA sequencing studies revealed a subpopulation of proliferative β-cells in rat islets that were cultured ex vivo in the presence of a high glucose concentration with the fatty acid oleate, as a means to mimic a diet with nutrient excess [110]. The selective proliferation of β-cells was confirmed by the absence of proliferating non-β-cell clusters. In the transcriptome of proliferating β-cells, an increased expression of pathways involved in oxidative phosphorylation and oxidoreductase activity was found, while β-cell maturation markers such as *Ins2*, *NeuroD1*, and *Pdx1* were downregulated.

In summary, processes related to oxidative metabolism are closely linked to the normal function of pancreatic β-cells, as these are directly involved in glucose sensing and insulin release. Although it is believed that both ROS and hypoxia-induced pathways have important regulatory functions in β-cells, single-cell omics research has also pointed out that oxidative stress is potentially harmful and could affect β-cell identity. Further research is needed to understand the exact contribution of oxidative stress to the pathophysiology of diabetes and to modulate its impact for potential therapeutic benefit.

## 6. Inflammation

In addition to ER stress and oxidative stress, inflammation plays a key role in diabetic complications. The three mechanisms are closely interconnected regarding their detrimental effects on β-cells. Inflammation entails the processes of recruitment, accumulation, and the activation of proinflammatory macrophages, along with the circulation of proinflammatory cytokines. Pancreatic islet inflammation is typically marked by infiltrating adaptive and innate immune effectors. Proinflammatory cytokines, such as interleukin-1β (IL-1β), interferon-γ (IFN-γ), and tumor necrosis factor-α (TNF-α), are considered important mediators of progressive β-cell destruction and dysfunction in both T1D and T2D [111,112]. When a sufficient amount of β-cell mass has been deemed nonfunctional and/or destroyed, blood levels will rise to a hyperglycemic state, and clinical diabetes is established. The inflammatory mechanisms involved in the development of β-cell dysfunction are different between T1D and T2D, as reflected in the distinct degrees of β-cell failure observed in both types of diabetes: in T1D, β-cell mass, and function are almost absent at diagnosis due to the increased concentration of cytotoxic T-cells and proinflammatory cytokines [113]. In contrast in T2D, the loss of β-cell mass and function occurs more slowly [114]. In addition, obesity and its ensuing metabolic stress and low-grade inflammation are known to have deleterious effects on β-cell function and have been proposed as important components in the development of β-cell failure in T2D [115].

As with ER stress and oxidative stress, there is a link between inflammation and β-cell identity loss. It has long been established that the prolonged exposure of primary rat β-cells to IL-1β leads to the repression of β-cell identity factors such as *Pdx1*, *Mafa*, and *Glut2*, while the mRNA expression level of the endocrine progenitor gene *c-Myc* is upregulated [116,117]. The treatment of different combinations of proinflammatory cytokines also leads to alterations in the β-cell-differentiated phenotype, as shown in isolated and purified rat β-cells after exposure to IL-1β with IFN-γ and to TNF-α with IFN-γ [118]. The isolation of human islets induces a strong inflammatory response that continues during in vitro culturing, manifested by the upregulation of several cytokines and cytokine receptors [60]. In addition, several pancreas-specific transcription factors were down-regulated in cultured islets, while pancreatic progenitor cell-specific transcription factors *SOX9*, *SOX4*, and *ID2* were upregulated, suggesting the loss of the mature endocrine cell phenotype. In cultured human and mouse islets, exposure to proinflammatory cytokines promotes β-cell dedifferentiation, as indicated by the downregulated gene expression of *PDX1*, *NKX6.1*, and *GLUT2* [119]. In particular, β-cell identity-maintaining factor FOXO1 was downregulated at the protein level upon IL-1β exposure. Furthermore, in vivo, anti-inflammatory treatment ameliorated the diabetes phenotype in mouse islets for some, but not all, proinflammatory cytokines. The fact that IL-1β has such a potent effect on β-cell identity and function could be the result of the particularly high expression level of the IL-1 receptor in β-cells [120]. In individuals without diabetes but with chronic pancreatitis, there was evidence found of dedifferentiated β-cells, suggesting that inflammation alone can induce β-cell identity loss [121]. Furthermore, in the same study there was an increased the expression of aldehyde dehydrogenase 1 family member A3 (ALDH1A3), which had been proposed as a marker of dedifferentiated β-cells [50].

Single-cell omics studies confirm the link between inflammation and β-cell identity change. Rui et al. described the development of a β-cell subpopulation during the progression of diabetes in NOD mice [122]. Using RNA-seq, the researchers showed that this subpopulation had a reduced expression of β-cell-specific genes like *Mafa*, *Nkx6.1*, and *Pdx1*, while the expression of endocrine progenitor-like genes such as *Neurog3*, *Sox9*, and MYCL proto-oncogene (*l-Myc*) were upregulated. Furthermore, this β-cell subgroup was less susceptible to immune attack through infiltrating lymphoid cells and proinflammatory cytokines by reducing the expression of diabetogenic antigens and increasing the expression of immunomodulatory molecules. The researchers propose that this adaptation may account for the chronicity of diabetes and the persistence of β-cells in T1D patients. In a comprehensive multi-omics study by Ramos-Rodriguez et al., it was shown that exposure of human β-cells to proinflammatory cytokines reveals a marked plasticity of the β-cell regulatory landscape [123]. By performing ATAC-seq and ChIP-seq, the researchers noted changes in chromatin accessibility upon the treatment of both human islet preparations and EndoC-βH1 cells with IFN-γ and IL-1β. A subset of these newly opened chromatin regions was preferentially located distally to gene transcription start sites and enriched for specific transcription factor binding sites. Further downstream, RNA-seq data and proteomics analysis indicated that the extensive gene regulatory activation led to the induction of transcription and protein translation pathways implicated in the pathogenesis of T1D. The exposure of isolated mouse islets to IL-1β under non-cytotoxic concentrations reduced insulin secretion, β-cell proliferation, and the expression of key β-cell identity genes, including *Mafa*, *Pdx1*, and *Ucn3* [124]. Although previously described in β-cell dedifferentiation [3,51], no nuclear–cytoplasmic shuttling of FOXO1 was observed in response to IL-1β, but there was a significant reduction in total FOXO1 protein. ChIP-seq data showed that histone modification marker H3K27ac, which was previously found to be associated with both active promotors and enhancers in human islet cells [125], was decreased at the gene loci, suggesting that inflammatory cytokines directly affect the epigenome. Upon the removal of IL-1β, β-cell function was normalized and the mRNA expression of some β-cell marker genes returned to pre-stimulation levels, indicating that inflammation-induced effects on β-cell identity might be reversible. In another scRNA-seq study conducted in isolated mouse islets, it was found that subsets of β-cells reacted differently to treatment with IL-1β and IFN-γ [126]. More specifically, only 70% of β-cells showed the increased expression of nitric oxide synthase 2 (*Nos2*), the gene transcribing an enzyme that produces nitric oxide, which was previously shown to protect β-cells from apoptosis through the inhibition of mitochondrial oxidation [127]. Although the researchers observed the repression of β-cell identity factors *Pdx1*, *Mafa*, *Nkx6.1*, and *Ucn3* after treatment with IL-1β, this did not coincide with the increased expression of β-cell disallowed genes or genes associated with dedifferentiation. Finally, in a Patch-seq setup, the expression of genes related to diminished β-cell exocytosis in T2D was associated with well-known controllers of inflammatory pathways, along with the transcription factor *ETV1* [128]. Knocking down *ETV1* in β-cells from donors with T2D resulted in the restoration of exocytosis capacity.

In summary, there is ample evidence that the occurrence of proinflammatory processes coincides with β-cell identity change. Single-cell omics studies uncovered specific subsets of β-cells after exposure to proinflammatory cytokines, resulting in varying degrees of β-cell identity change. One caveat of studies focused on inflammation is the fact that, in most cases, just one or two inflammatory cytokines are used to induce β-cell dysfunction, as opposed to the exposure of islet cells to high glucose and other adverse inflammatory and/or metabolic factors in individuals with diabetes. In addition, the exact level of inflammatory mediators in the local in vivo situation remains uncertain. Furthermore, the build-up to β-cell dysfunction is a multi-faceted process that cannot be attributed to inflammation alone. It is rather the outcome of a complex interplay between chronic hyperglycemia, glucolipotoxicity, and several other metabolic processes like ER stress, oxidative stress, and inflammation, as shown for example by several studies that reported an ER stress response in β-cells upon treatment with inflammatory mediators [129,130,131,132]. One of the goals for single-cell omics studies may be to distinguish these processes from one another and map their downstream pathways in more detail to target them more efficiently.

## 7. Concluding Remarks

**Figure 3:**
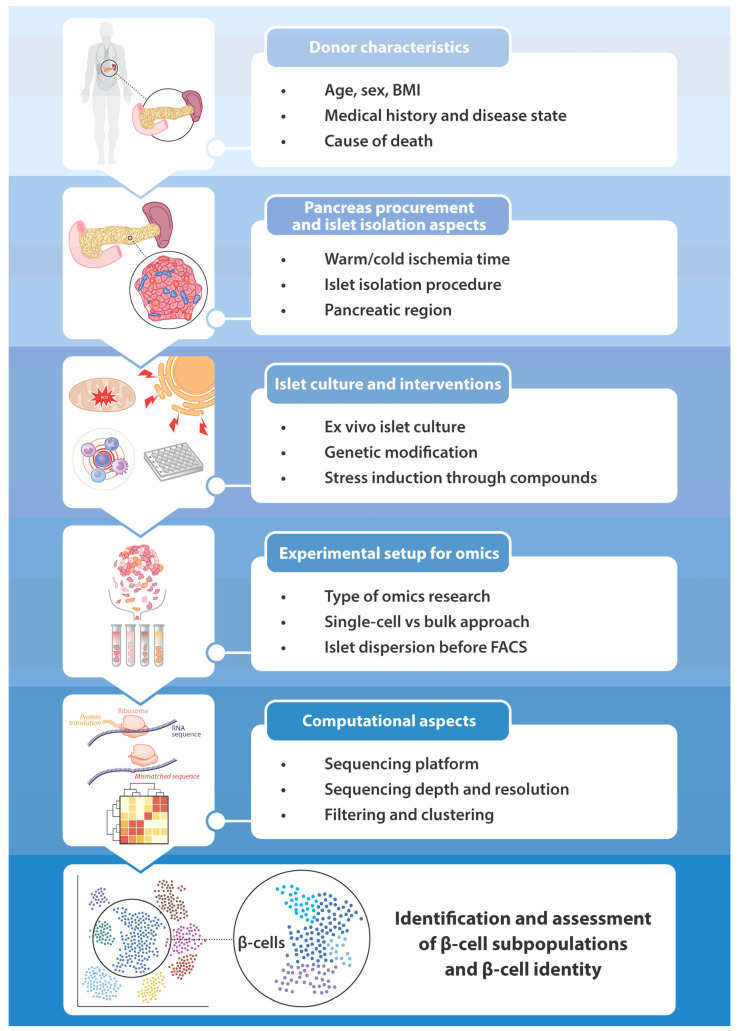
Factors that may influence the outcome of a single-cell omics study on β-cell identity.

The development of single-cell omics techniques has led to an unprecedented breakthrough for characterizing the transcriptome, epigenome, and proteome of islet cell types in general and β-cells in particular. Several important observations on the regulation of β-cell-specific gene and protein expression have been made in the past decade. Multiple studies have identified differentially expressed genes due to altered metabolic states such as diabetes mellitus or the activation of ER stress-, oxidative stress-, and inflammation-induced pathways. However, as discussed in this review, it is important to keep in mind that several factors in the design of a single-cell omics experiment can influence the assessment of β-cell identity. Those are summarized in Figure 3. Before starting the experiment, donor characteristics have to be taken into account. These include age, sex, BMI, medical history, disease state, and cause of death. Furthermore, pancreas procurement and islet isolation aspects are relevant. These could be the duration of the warm or cold ischemia time, the exact islet isolation procedure, and the pancreatic regions that were processed. In addition, the method of preparing the tissue for the experiment plays a role in the outcome, such as the conditions of the ex vivo islet culture, any genetic modification, and the addition of compounds to induce changes in cell identity and function. Important elements in the experimental setup are determining what type of omics research will be conducted (transcriptomics, epigenomics, proteomics, etc.), whether a single-cell or a bulk method is the most suitable approach, and the fact that islets are dispersed prior to cell sorting. The last consideration, especially in the field of single-cell omics research, is about computational aspects. These include choosing the appropriate sequencing platform, picking the right sequencing depth to reach the desired resolution, and determining the stringency of filtering and the parameters for clustering. Genes expressed at low levels may not be detected, noise may not be appropriately filtered, and there could be selection bias for certain cell types. Further advancements in single-cell omics methods and the development of new computational tools, also involving AI (artificial intelligence) and machine learning, could eventually overcome these issues. The big challenge is to utilize the full potential of single-cell omics techniques to increase our understanding of islet biology and, more specifically, β-cell identity change.

## Figures and Tables

**Table 1:** Summary of main findings in omics studies focusing on β-cell heterogeneity and β-cell identity.

Topic	Species	Method	Identification of β-Cell Heterogeneity Based on:	Ref.
β-cell heterogeneity	Mouse	bulkRNA-seq	Expression of immature β-cell gene panel and lacking UCN3	[20]
		scRNA-seq	Expression of transcription factor genes *Srf*, *Jun*, and *Fos*	[21]
		SCAN-seq ChIP-seq	Heterogeneity in the β-cell epigenome	[22]
	Human	scRNA-seq	Presence of cell surface markers ST8S1A1 and CD9	[23]
		scRNA-seq	Expression of transcriptional regulators *RBP4*, *FFAR4*, *ID1*, *ID2*, and *ID3*	[25]
		scRNA-seq	Expression of oxidative stress-implicated gene *FTH1*	[29]
		scRNA-seq	Expression of ER stress-inducible genes *HERPUD1*, *HSPA5*, and *DDIT3*	[30]
		snATAC-seq	Heterogeneity in the β-cell epigenome	[31]
		scRNA-seq	Expression of novel transcriptional states related to stress	[33]
		snRNA-seq scRNA-seq	Expression of *INS* mRNA levels	[34]
		scRNA-seq	Expression of cell surface marker CD63	[35]
β-cell identity in diabetes	Human	scRNA-seq	Expression of immature β-cell gene panel in islets from T2D donors	[38]
		scRNA-seq	De-repression of juvenile gene sets in β-cells from T2D donors	[42]
		scRNA-seq	Reduced expression of *INS* mRNA levels in β-cells from T2D donors	[25]
		bulkRNA-seq proteomics	Expression of β-cell precursor marker ALDOB in islets from T2D donors	[43]
		scRNA-seq snATAC-seq	Reduced expression of *HNF1A* in β-cells from T2D donors	[44]
		scRNA-seq	Transcriptional states in β-cells from T1D donors	[45]
		scRNA-seq	Transcriptional states in β-cells from T1D and T2D donors	[46]
